# The Role of Cardiovascular Magnetic Resonance Imaging in the Assessment of Myocardial Fibrosis in Young and Veteran Athletes: Insights From a Meta-Analysis

**DOI:** 10.3389/fcvm.2021.784474

**Published:** 2021-12-21

**Authors:** Emmanuel Androulakis, Dimitrios Mouselimis, Anastasios Tsarouchas, Alexios Antonopoulos, Constantinos Bakogiannis, Panagiotis Papagkikas, Charalambos Vlachopoulos

**Affiliations:** ^1^Royal Brompton Hospital, Imaging Centre, Cardiac Magnetic Resonance Unit, London, United Kingdom; ^2^Third Department of Cardiology, Aristotle University of Thessaloniki, Thessaloniki, Greece; ^3^Unit of Inherited Cardiac Conditions, First Cardiology Department, University of Athens, Athens, Greece

**Keywords:** athletes, myocardial fibrosis, LGE, CMR, meta-analysis, mapping

## Abstract

**Background:** Cardiac magnetic resonance (CMR) combined with late gadolinium enhancement (LGE) has revealed a non-negligible increased incidence of myocardial fibrosis (MF) in athletes compared to healthy sedentary controls.

**Objective:** The aim of this systematic research and meta-analysis is to investigate and present our perspective regarding CMR indices in athletes compared to sedentary controls, including T1 values, myocardial extracellular volume (ECV) and positive LGE indicative of non-specific fibrosis, also to discuss the differences between young and veteran athletes.

**Methods:** The protocol included searching, up to October 2021, of MEDLINE, EMBASE, SPORTDiscus, Web of Science and Cochrane databases for original studies assessing fibrosis via CMR in athletes. A mean age of 40 years differentiated studies' athletic populations to veteran and young.

**Results:** The research yielded 14 studies including in total 1,312 individuals. There was a statistically significant difference in LGE fibrosis between the 118/759 athletes and 16/553 controls (*Z* = 5.2, *P* < 0.001, *I*^2^ = 0%, *P*_I_ = 0.45). Notably, LGE fibrosis differed significantly between 546 (14.6%) veteran and 140 (25.7%) young athletes (*P* = 0.002). At 1.5T, T1 values differed between 117 athletes and 48 controls (*P* < 0.0001). A statistically significant difference was also shown at 3T (110 athletes vs. 41 controls, *P* = 0.0004), as well as when pooling both 1.5T and 3T populations (*P* < 0.00001). Mean ECV showed no statistically significant difference between these groups.

**Conclusions:** Based on currently available data, we reported that overall LGE based non-specific fibrosis and T1 values differ between athletes and sedentary controls, in contrast to ECV values. Age of athletes seems to have impact on the incidence of MF. Future prospective studies should focus on the investigation of the underlying pathophysiological mechanisms.

## Introduction

Athletic training is known to induce morphological and functional cardiovascular adaptations of cardiac chambers, collectively known as athlete's heart (1). Apart from the widely established benefits of exercise, recent evidence suggests there may be some overlap between physiology and pathophysiology, and occasionally, with cardiac diseases. The duration of exposure to demanding training and the type of sports may play a role in these processes (2). Cardiac magnetic resonance (CMR) has been increasingly used in establishing an accurate diagnosis given that exercise may lead to cardiac remodeling that in certain situations can be clinically challenging to differentiate from various cardiomyopathies (3, 4).

CMR is a non-radiating imaging modality with high spatial resolution, which not only is the reference standard for functional and morphological assessment but also has the benefit of tissue characterization by exploiting gadolinium-based contrast late enhancement (LGE) as a marker of myocardial fibrosis (MF) (5, 6). Gadolinium-based contrast compounds freely enter the extracellular space but not intact myocardial cells. Under pathologic conditions, cellular death and fibrotic processes lead to expansion of extracellular space, while cell damage means that myocardial cell membranes become permeable to contrast. These phenomena significantly alter the kinetics of gadolinium, leading to higher peak uptake from the myocardium and delayed washout (7). Interestingly specific patterns of LGE have been sporadically detected in athletic individuals, although data so far are not consistent, coming from small-sample studies that frequently lack comparisons with sedentary controls or “lifelong,” veteran athletic individuals which could provide further insight in the underlying pathophysiological mechanisms (8).

Other important techniques, such as native T1 and extracellular volume (ECV) may prove clinically relevant in athletic individuals providing additional information regarding cellular and extracellular pathology, even though they have not yet been widely studied in these populations (9). Native T1 times quantify the time required for the net magnetization vector of a myocardial area to return to pre-excitation levels. Native T1 depends highly on the tissue composition (10). Elevated T1 times have been reported in several myocardial pathologic conditions. Pertinent to the topic of this review, T1 values increase in situations of increased free water within expanded interstitial space and could be used to detect and quantify interstitial myocardial fibrosis (11). The measurement of pre- and post-contrast T1 times enable the CMR-based calculation of ECV with good correlation to the actual histology-derived collagen volume fraction (12). The ratio of intra- to extracellular space shifts significantly in myocardial fibrosis, and ECV is uniquely capable of detecting such changes. It thus constitutes an ideal modality for the clinical applications described in the present review (13).

## Systematic Review

### Systematic Review Rationale-Objectives

In a previous systematic review involving only 65 athletes (14), van de Schoor et al. predominantly identified MF in the intraventricular septum and the right ventricular (RV) insertion points. Although the underlying mechanisms are widely undetermined, the summarized evidence supported genetic predisposition, silent myocarditis, pulmonary artery pressure overload, and prolonged exercise-induced repetitive micro-injury as possible contributors (15). More recently, Zhang et al. (16) performed a meta-analysis of athletic individuals and sedentary controls who underwent CMR, focusing however only on general MF prevalence without looking into different patterns, and without discriminating different athletic age groups, or sex-specific data. According to the results, 21.1% of athletes had evidence of LGE, compared to just 3.2% in sedentary controls. The heterogeneity of the 12 included studies was acceptable (*P* = 0.34), while the difference in prevalence was statistically significant, suggesting a correlation between MF and intense athletic training.

In view of updated data on the topic of CMR based assessment, using both contrast and non-contrast techniques, we performed a revised systematic search and meta-analysis, with a three-fold aim: To focus on updated, peer-reviewed data, report a risk of bias assessment, which was unfortunately missing from the recent meta-analysis (16), and extract data on other CMR-derived techniques, such as native T1 mapping as a sensitive marker of interstitial fibrosis and ECV quantification, as marker of myocardial tissue remodeling, based on studies using both 1.5 and 3 Tesla (T) scanners. Of note, structured assessment of risk of bias and exclusion of low-quality data is crucial, since recruitment bias can have an enormous effect on the prevalence of LGE in an athlete population.

### Systematic Review Strategy

The systematic research protocol was registered in the PROSPERO database (ID: CRD42021273996). MEDLINE, EMBASE, SPORTDiscus, Web of Science and Cochrane databases were systematically searched with the use of PubMed, Google Scholar and Cochrane Reviews search Engines between January 2000 up to October 2021. Studies were eligible for inclusion in the systematic review if they evaluated one or more of the following parameters in endurance sports athletes: (a) The presence of late gadolinium enhancement, (b) Native T1 values and (c) ECV. The Oxford Dictionary definition was used to identify endurance sports (“a sport that involves continuous high intensity exercise”) (17). Only studies reported in English were assessed for inclusion. As for the exclusion criteria, studies lacking a control arm of age- and sex-matched individuals were excluded from the systematic review, as were studies in which either controls or athletes had been included on the basis of having symptoms or signs of cardiac pathology (e.g., premature ventricular contractions) were excluded. When multiple studies reported on data from the same research group, only one was kept, unless it is explicitly stated that there was no overlap. A detailed presentation of the systematic protocol is described in Supplementary Material. Bias assessment was performed via the Newcastle-Ottawa quality assessment scale (NOS) for cohort studies (18). The Review Manager (RevMan) Version 5.3 and SPSS (IBM Corp. Released 2015. IBM SPSS Statistics for Windows, Version 23.0. Armonk, NY: IBM Corp.) were used for statistical analysis. Random effect was the model of choice for all pooled analyses with Z-value for overall effect and *I*^2^ for heterogeneity. Heterogeneity was considered significant at the level of *p* < 0.10. T1 and ECV, were separately assessed for 1.5 and 3 Tesla and afterwards in total. The incidence of LGE in younger vs. veteran athletes (cut-off set at 40y) was compared in a sub-analysis through the chi-square test. Reported means and standard deviations (SD) are pooled from study data. Data was mathematically transformed if needed. *P*-values of <0.05 were considered statistically significant unless otherwise stated.

## Results

### LGE in Athletes and Sedentary Controls

**Figure 1:**
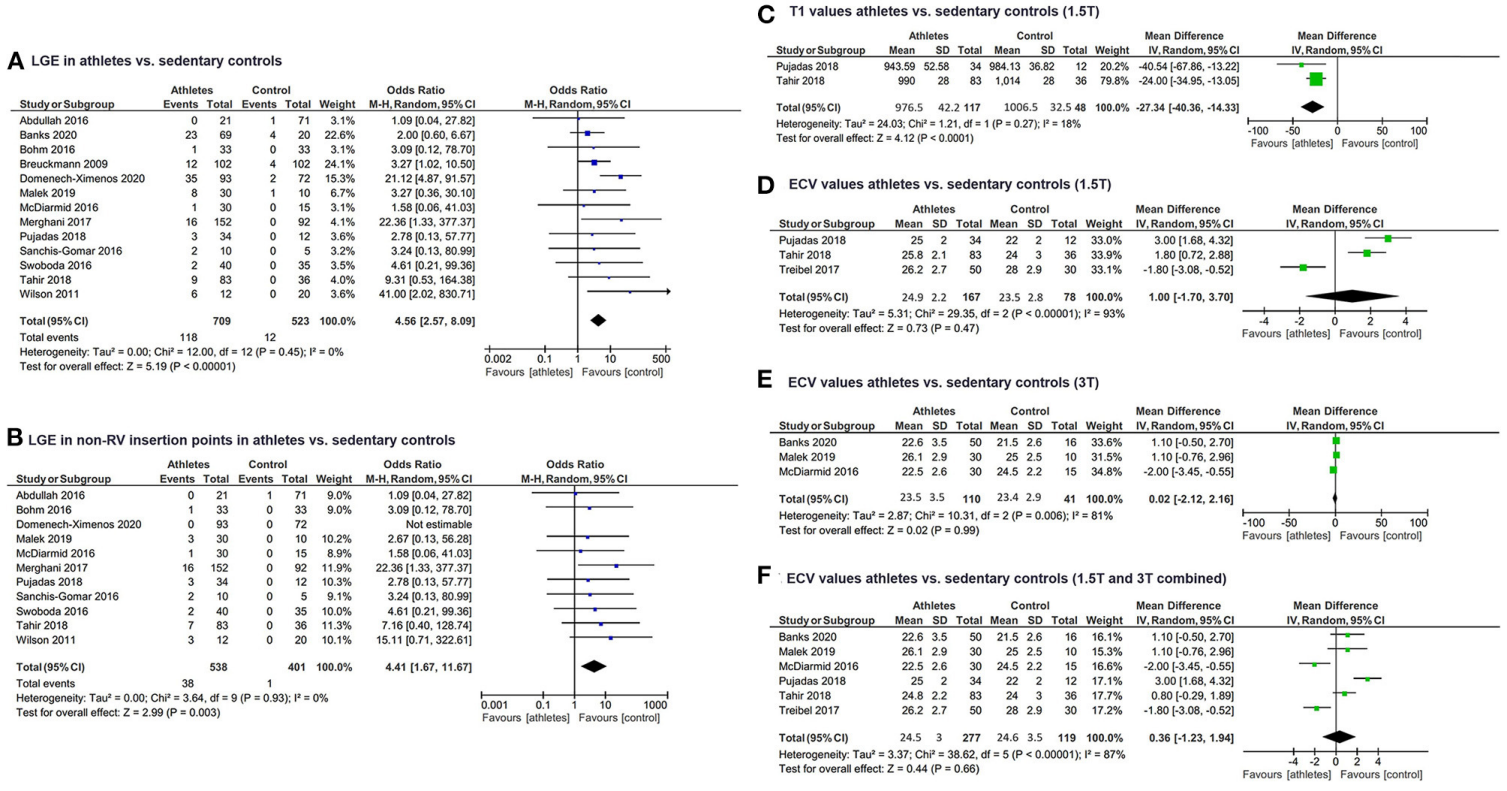
Forest plots of **(A)** LGE prevalence in athletes and sedentary controls, **(B)** LGE prevalence excluding RV insertion point LGE in athletes and sedentary controls, **(C)** Native T1 values from 1.5T CMR scans in athletes and sedentary controls, **(D)** ECV from 1.5T CMR scans in athletes and sedentary controls, **(E)** ECV from 3T CMR scans in athletes and sedentary controls, **(F)** Pooled ECV in athletes and sedentary controls. LGE, late gadolinium enhancement; RV, right ventricle; CMR, cardiac magnetic resonance; ECV, extracellular volume.

Fourteen (14) studies (4, 19–31) matching the pre-specified inclusion criteria were found and included in the updated review and meta-analysis (Table 1). A risk of bias assessment was performed using the Newcastle Ottawa Scale (Supplementary Table 1). In total, 759 athletes and 553 sedentary controls were included in the updated meta-analysis. Out of these, 118 (16.6%) athletes and 12 (2.3%) controls had LGE, a difference in proportions that proved statistically significant (*Z* = 5.2, *P* < 0.001). Study heterogeneity regarding LGE was low (*I*^2^ = 0%, *P*_I_ = 0.45) (Figure 1). Most of the studies either included only male athletes and controls or reported sex-specific data. Forest plots could be constructed for male (4, 21, 22, 24–29, 31) and female (21, 22, 31) athletes. In total, LGE data regarding 460 male athletes were compared to those of 315 sedentary controls. The Z-overall effect was estimated at 4.76 with *P* < 0.001. Heterogeneity was low (*I*^2^ = 0%, textitP_I_ = 0.80). Regarding females, 119 athletes were compared with 82 sedentary controls. No statistically significant difference was observed (*P* = 0.10) for a Z-overall effect of 1.67, while heterogeneity was not negligible (*I*^2^ = 44%, *P*_I_ = 0.18).

**Table 1:** Characteristics of the studies included in the meta-analysis.

	**CMR findings**		**Athletes**	**Characteristics**	
**Studies Tesla (T)**	**Athletes**	**Controls**	**Age and sex**	**Athletes group**	**Control group**
Abdullah et al. 2016 (19) T: 1.5	LGE: 0/21 T1: No data ECV: No data	LGE: 1/71 T1: No data ECV: No data	68 (66–70), 76.2% males	Elite marathon and triathlon athletes with 6–7 30 min sessions per week for ≥25 years *Fibrosis: No data*	No statistical difference for age; sedentary to light athleticism *Fibrosis: One IVS in the area of the inferior RV insertion point*
Banks et al. 2020 (20) T: 3	LGE: 23/69 T1 (*n* = 50): 1169 ± 35 ECV (*n* = 50): 22.6 ± 3.5	LGE: 4/20 T1 (*n* = 16): 1190 ± 26	53 ± 5, 74% males (for 72 athletes)	Middle-aged endurance running (24), cycling (20), and triathlon (28) athletes with <10 years of active participation in competitive sport competitions *Fibrosis (athletes and controls combined): 21/89 RV insertion points, 2/89 ischemic and 4/89 with no ischemic etiology*	Mildly active according to recommendations *Fibrosis: see athletes' fibrosis*
Bohm et al. 2016 (4) T: 1.5	LGE: 1/33 T1: No data ECV: No data	LGE: 0/33 T1: No data ECV: No data	47 ± 8, 100% males	Veterans with 16 former elite athletes; triathlon, ironman, Olympics (triathlon and rowing), marathon training >10 h per week for >10 years (29 ± 8 years) *Fibrosis: One posteroinferior visible in the short axis following a non-ischemic pattern*	Age, height and weight matched; exercise history of ≤ 3 hours per week; *Fibrosis: No data*
Breuckman et al. 2009 (25) T: 1.5	LGE: 12/102 T1: No data ECV: No data	LGE: 4/102 T1: No data ECV: No data	57 ± 6, 100% males	Athletes over 50 years old having participated in at least five full-distance marathons in the last 3 years *Fibrosis: Five with CAD pattern affecting segments 10 in the region of LAD, one of LCA, three RCA vs. five with no-CAD pattern affecting three of LAD, five of LCA and nine of RCA*	Age matched controls; no endurance sports activity *Fibrosis: Four having no CAD-pattern affecting 0 segments of the LAD, three of LCA and six of RCA*
Domenech-Ximenos et al. 2020 (21) T: 1.5, 3	LGE: 35/93 T1: No data ECV (*n* = 28, 1.5T): LGE (+) 27.1 ± 2.2 vs. LGE (–) 25.2 ± 2.1	LGE: 2/72 T1: No data ECV: No data	35.7 ± 5.8, 53% males	Triathlon athletes with >12 h per week active in the last 5 yearsr *Fibrosis: 35 RV insertion points (17/49 males and 18/44 females)*	Age and sex matched; <3 h of training per week *Fibrosis: Two RV insertion points (only in males)*
Malek et al. 2019 (26) T: 3	LGE: 8/30 T1: 1200 ± 59 ECV: 26.1 ± 2.9	LGE: 1/10 T1: 1214 ± 32	40.9 ± 6.6 ECV: 25 ± 2.5, 100% males	Ultra-marathon runners with a median of 9 years of regular event competing *Fibrosis: Five RV insertion point, two inferolateral, one IVS, none with ischemic pattern*	Age and sex matched; no regular exercising *Fibrosis: One RV insertion points, none with ischemic pattern*
McDiarmid et al. 2016 (27) T: 3	LGE: 1/30 T1: 1178 ± 32 ECV: 22.5 ± 2.6	LGE: 0/15 T1: 1202 ± 33 ECV: 24.5 ± 2.2	31.7 ± 7.7, 100% males	Endurance athletes (seven runners, 11 cyclists, 12 thriathletes) training for >6 h per week *Fibrosis: One following myocarditis pattern*	Age and sex matched; no endurance sports activity with <3 h training per week *Fibrosis: No data*
Merghani et al. 2017 (22) T: 1.5	LGE: 16/152 T1: No data ECV: No data	LGE: 0/92 T1: No data ECV: No data	54.4 ± 8.5, 70% males and 92% reported as “white”	Masters running and cycling athletes who have run ≥10 miles or cycled ≥30 miles per weak and competed frequently for >10 years in at least 10 endurance events *Fibrosis: 10 basal lateral or inferolateral (nine were men), four septal, two apical, Athletes and controls: subendocardial in seven males, midmyocardial in five and epicardial distribution in three; Only 1 female athlete had LGE*	Age, sex, and 10 year Framingham risk score close to the athletes group; mildly trained according to health recommendations *Fibrosis: No data*
Pujadas et al. 2018 (28) T: 1.5	LGE: 3/34 T1 (septal): 943.59 ± 52.58 ECV (septal): 25 ± 2	LGE: 0/12 T1 (septal): 984.13 ± 36.82 ECV (septal): 22 ± 2	48.17 ± 7.48, 100% males	Veteran marathon still training having participated in marathons for >10 years (9.38 ± 3.52 h of training per week, 28.06 ± 10.84 years of training) *Fibrosis: One mid inferior, one mid inferolateral, one apical (antero)septum, none with ischemic pattern*	Age, sex and BSA matched; untrained *Fibrosis: No data*
Sanchis-Gomar et al. 2016 (29) T: 3	LGE: 2/10 T1: No data ECV: No data	LGE: 0/5 T1: No data ECV: No data	Elite: 54 ± 4, Sub-elite: 55 ± 9, 100% males, not applicable for those who underwent CMR	11 elite (10.6 ± 3.1 h per week, 29 ± 9 years high-intensity trained) and 42 sub-elite (10.6 ± 4.2 h per week, 24 ± 9 years high-intensity trained) endurance athletes (cyclists and runners). Only 10 (3 were elite and the remaining were sub-elite) underwent CMR *Fibrosis: 1 intra-myocardial fibrotic lession in the LV lateral wall, 1 small intra-myocardial in the basal segment of the inferolateral LV wall. None had ischemic pattern*	Age and sex matched; <3 structured training sessions per week. Only five underwent CMR. *Fibrosis: No data*
Swoboda et al. 2016 (30) T: 3	LGE: 2/40 T1: 1182.7 ± 42.4 ECV: 22.7 ± 3.3	LGE: 0/35 T1: No data ECV: 24.3 ± 2.6	<45 years, no sex information	Endurance athletes (11 runners, 13 triathletes, 16 cyclists) with >6 h per week *Fibrosis: Two subepicardial lateral with a pattern of myocarditis*	<3 h of training per week *Fibrosis: No data*
Tahir et al. 2018 (31) T: 1.5	LGE: 9/83 T1: 990 ± 28 ECV: 25.8 ± 2.5	LGE: 0/36 T1: 1014 ± 28 ECV: 25.9 ± 3.9	43 ± 10, 65% males	Triathlon athletes with >10 h per week active in the last 3 years *Fibrosis (only males): Six mid-wall basal inferolateral, two posterior RV insertion, one basal anterolateral subendocardial all without ischemic pattern*	<3 h of exercise per week *Fibrosis: No data*
Treibel et al. 2017 (23) T: 1.5	LGE: No data T1: No data ECV (*n* = 50): 26.2 ± 2.7	LGE: No data T1: No data ECV (*n* = 30): 28 ± 2.9	42 ± 14, 80% males	Endurance athletes with >10 events in lifetime *Fibrosis: No data*	No statistical difference for age *Fibrosis: No dat*a
Wilson et al. 2011 (24) T: 1.5	LGE: 6/12 in Veteran, 0/17 in Young T1: No data ECV: No data	LGE: 0/20 T1: No data ECV: No data	(Veterans)57 ± 6 (50–67)/(Young)31 ± 5 (26–40), 100% males	Marathon, ultramarathon, ironman, and triathlon veteran (43 ± 6 years of competitive training) and young (18 ± 7 years competitively trained) athletes *Fibrosis (only veterans): Three RV insertion points, one subendocardial septal and lateral wall with CAD pattern, one subepicardial lateral, one mid-wall mid-apical inferior*	Age matched with veteran athletes; sedentary lifestyle *Fibrosis: No data*

*CMR, cardiac magnetic resonance; LGE, late gadolinium enhancement; ECV, extracellular volume; min, minutes; RV, right ventricular; CAD, coronary artery disease; LAD, left anterior descending artery; LCA, left coronary artery; RCA, right coronary artery; IVS, interventricular septum*.

Regarding the comparison of young vs. veteran athletes, LGE data were available for 10 studies. A mean age over 40 years (546 athletes total, 14.6% exhibited LGE) and three studies with a mean age below 40 years (140 athletes total, 25.7% exhibited LGE). The chi-square test revealed a statistically significant difference of LGE incidence between the two groups of veteran and young athletes (chi-square = 9.7, *P* = 0.002).

LGE in RV insertion points is increasingly considered a non-specific finding of unknown significance (21, 22). A focused sub-analysis investigating the incidence of non-RV insertion point LGE revealed that the prevalence is also substantially higher in athletes (38 out of 538 included, 7%) compared to controls (one out of 401 included, 0.3%, *Z* = 2.99, *P* = 0.003).

### T1 and ECV in Athletes vs. Sedentary Controls

Data regarding alternative non-contrast markers such as T1 and ECV derived from 3T and 1.5T MRI were extracted separately from included studies. Two studies (28, 31) had 1.5T T1 data available for a total of 117 athletes (mean T1 976.5 ± 42.2 msec) and 48 controls (1006.5 ± 32.5 msec) showed a statistically significant difference (*Z* = 4.12, *P* < 0.0001, *I*^2^ = 18%, *P*_I_ = 0.27). T1 data with 3T scanners were similar—three studies (20, 26, 27) including 110 athletes (mean T1 1179.9 ± 42.1 msec) and 41 controls (mean T1 1200.2 ± 31 msec) also yielded a statistically significant difference in T1 values between athletes and sedentary controls (*Z* = 3.55, *P* = 0.0004, *I*^2^ = 0%, *P*_I_ = 0.86) (Figure 1). When pooled into one forest plot, data from 1.5T and 3T CMR scans revealed a significant difference in T1 values between the two groups, while having no heterogeneity (*Z* = 6.15, *P* < 0.00001, *I*^2^ = 0%, *P*_I_ = 0.66). Controls' mean T1 was estimated at 1095.7 ± 101.7 ms, whereas athletes' mean T1 was 1075.1 ± 110.4 msec.

ECV quantified via 1.5T MRI scanners (23, 28, 31) in three studies did not differ significantly (*Z* = 0.73, *P* = 0.47, *I*^2^ = 93%, *P*_I_ = *P*_I_ < 0.00001) between the total 167 athletes (mean ECV 24.9 ± 2.2%) and 78 sedentary controls (mean ECV 23.5 ± 2.8%). MRI scans at 3T (20, 26, 27) comparing ECV between 110 athletes (mean ECV 23.5 ± 3.5%) to 41 sedentary controls (mean ECV 23.4 ± 2.9%) were also characterized by a not statistically significant mean difference (*Z* = 0.02, *P* = 0.99, *I*^2^ = 81%, *P*_I_ < 0.006) (Figure 1). The same tendency was observed when pooling mean ECV of athletes (24.5 ± 3%) and control groups (24.6 ± 3.5%) scanned with 1.5T and 3T and comparing them, as the mean difference had a *Z* = 0.42 and *P* = 0.66 (I^2^ = 87%, *P*_I_ < 0.00001).

## Discussion

All available evidence indicates that the prevalence of MF, as documented by LGE in CMR scans, is significantly higher in athletes compared to sedentary controls. The pooled frequency of 16.6% is lower than the 21.1% reported by Zhang et al. (16), with the difference owed to the updated set of studies, which include new data, updated numbers from some research teams and exclude some previous, non-peer-reviewed datasets.

In a close examination of the included studies, LGE was indeed considerably more frequent in athletes compared to controls, even when excluding RV insertion point fibrosis. Studies that included younger athletes also had a significantly higher prevalence of LGE compared to studies including veteran athletes. This is an interesting finding that is open to interpretation. It is not inconceivable that the differences in recruitment strategy between studies that led to an age disparity also caused differences in the prevalence of LGE. Importantly, Domenech-Ximenos et al., one of the included studies with the lowest mean athlete age, only administered gadolinium-based contrast to a sub-set of participating patients, potentially introducing bias in LGE results (21). Overall, the long-term effects of endurance exercise on the heart are widely unknown. Endurance exercise is associated with a transient increase of biomarkers of cardiac damage and there is growing evidence that lifelong male athletes aged above 40 years show a higher prevalence of a higher coronary plaque burden, and a different MF pattern compatible with subclinical infarction compared with relatively sedentary healthy controls (32). As no adequate data were available for females, no sex-specific conclusions could be safely drawn from our analysis. Further, ideally prospective studies with sizeable athlete populations are required to determine the effect of duration exercise on the incidence, pattern, and extent of MF as well as its prognostic relevance.

Quite interestingly, native T1 values were consistently shown to be significantly decreased in athletes, both in 1.5T and 3T magnetic fields. It has been suggested that in athletic left ventricular hypertrophy (LVH), native T1 seems to be decreased suggesting that physiological athletic LVH represents enhanced cellular hypertrophy, unlike any other sort of LVH mechanism (6). The ECV as a marker of myocardial tissue remodeling and excessive collagen deposition is also a robust measure of diffuse MF and fairly interesting from a pathophysiological perspective. It would not be unreasonable to assume that, in the setting of exercise-induced cardiomyocyte hypertrophy (6), the ECV is expected to be reduced. That said, the ECV was not found significantly increased in athletes compared to controls, and indeed some studies yielded the opposite result (23, 27). Whether this is the result of study selection bias, relatively small numbers of included so far studies, or whole-heart sub-clinical expansion of ECV through increased collagen deposition in maladapted athletes' hearts (33) is an interesting research question that deserves further investigation.

In conclusion, we report interesting data based on the latest evidence in MF assessment from both contrast based and non-contrast CMR techniques. The present systematic review and meta-analysis contains updated data about LGE prevalence along with a comprehensive risk of bias assessment, in combination with a novel meta-synthesis of data regarding T1 and ECV values in the endurance athletes. Non-specific MF in athletic individuals is a somewhat frequent finding in highly trained athletes and there seem to be variation attributable to age (and therefore potentially the duration of exposure to exercise) and sex. Contrast based non-specific LGE and native T1 are found to be particularly useful in discrimination of athletic vs. sedentary individuals. Further data are certainly required to elucidate the underlying physiological and pathophysiological mechanisms. More importantly, whether these non-specific LGE patterns in athletes are actually associated with adverse events-particularly for underrepresented athlete groups such as women and veteran athletes, as well as the effect of deconditioning on the fibrotic process, are all certainly topics for further research.

## Data Availability Statement

The raw data supporting the conclusions of this article will be made available by the authors, without undue reservation.

## Author Contributions

EA and CV conceived and designed the present systematic review. AA and PP also contributed meaningfully to the design of the systematic review and meta-analysis. DM, AT, and CB performed the systematic literature search. DM, AT, CB, and AA performed the analysis of data. EA, DM, AT, and CB wrote the initial draft versions of the manuscript. PP, AA, and CV performed significant revisions. All authors provided critical feedback and helped shape the research, analysis, and manuscript.

## Conflict of Interest

The authors declare that the research was conducted in the absence of any commercial or financial relationships that could be construed as a potential conflict of interest.

## Publisher's Note

All claims expressed in this article are solely those of the authors and do not necessarily represent those of their affiliated organizations, or those of the publisher, the editors and the reviewers. Any product that may be evaluated in this article, or claim that may be made by its manufacturer, is not guaranteed or endorsed by the publisher.

## Abbreviations

CMR: cardiac magnetic resonance
LGE: late gadolinium enhancement
ECV: extracellular volume
min: minutes
RV: right ventricular
CAD: coronary artery disease
LAD: left anterior descending artery
LCA: left coronary artery
RCA: right coronary artery
IVS: interventricular septum.

## References

[1] Sharma S Merghani A Mont L. Exercise and the heart: the good, the bad, the ugly. Eur Heart J. (2015) 36:1445–53. 10.1093/eurheartj/ehv090 25839670

[2] La Gerche. Can intense endurance exercise cause myocardial damage and fibrosis? Curr Sports Med Rep. (2013) 12:63–9. 10.1249/JSR.0b013e318287488a 23478555

[3] Gati S Sharma S Pennell D. The role of cardiovascular magnetic resonance imaging in the assessment of highly trained athletes. JACC Cardiovasc Imaging. (2018) 11(2 Pt 1):247–59. 10.1016/j.jcmg.2017.11.016 29413645

[4] Bohm P Schneider G Linneweber L Rentzsch A Kramer N Abdul-Khaliq H. Right and left ventricular function and mass in male elite master athletes: a controlled contrast-enhanced cardiovascular magnetic resonance study. Circulation. (2016) 133:1927–35. 10.1161/CIRCULATIONAHA.115.020975 27073129

[5] La Gerche A Taylor AJ Prior DL. Athlete's heart: the potential for multimodality imaging to address the critical remaining questions. JACC Cardiovasc Imaging. (2009) 2:350–63. 10.1016/j.jcmg.2008.12.011 19356581

[6] Maestrini V Torlasco C Hughes R Moon JC. Cardiovascular magnetic resonance and sport cardiology: a growing role in clinical dilemmas. J Cardiovasc Transl Res. (2020) 13:296–305. 10.1007/s12265-020-10022-7 32436168 PMC7360536

[7] Doltra A Amundsen BH Gebker R Fleck E Kelle S. Emerging concepts for myocardial late gadolinium enhancement MRI. Curr Cardiol Rev. (2013) 9:185–90. 10.2174/1573403X113099990030 23909638 PMC3780343

[8] Androulakis E Swoboda PP. The role of cardiovascular magnetic resonance in sports cardiology; current utility and future perspectives. Curr Treat Options Cardiovasc Med. (2018) 20:86. 10.1007/s11936-018-0679-y 30167977 PMC6132733

[9] Robinson AA Chow K Salerno M. Myocardial T1 and ECV measurement: underlying concepts and technical considerations. JACC Cardiovasc Imaging. (2019) 12(11 Pt 2):2332–44. 10.1016/j.jcmg.2019.06.031 31542529 PMC7008718

[10] Moon JC Messroghli DR Kellman P Piechnik SK Robson MD Ugander M. Myocardial T1 mapping and extracellular volume quantification: a Society for Cardiovascular Magnetic Resonance (SCMR) and CMR Working Group of the European Society of Cardiology consensus statement. J Cardiovasc Magn Reson. (2013) 15:92. 10.1186/1532-429X-15-92 24124732 PMC3854458

[11] Ferreira VM Piechnik SK Robson MD Neubauer S Karamitsos TD. Myocardial tissue characterization by magnetic resonance imaging: novel applications of T1 and T2 mapping. J Thorac Imaging. (2014) 29:147–54. 10.1097/RTI.0000000000000077 24576837 PMC4252135

[12] Fontana M White SK Banypersad SM Sado DM Maestrini V Flett AS. Comparison of T1 mapping techniques for ECV quantification. Histological validation and reproducibility of ShMOLLI versus multibreath-hold T1 quantification equilibrium contrast CMR. J Cardiovasc Magn Reson. (2012) 14:88. 10.1186/1532-429X-14-88 23272651 PMC3552758

[13] Bakogiannis C Mouselimis D Tsarouchas A Papatheodorou E Vassilikos VP Androulakis E. Hypertrophic cardiomyopathy or athlete's heart? a systematic review of novel cardiovascular magnetic resonance imaging parameters. Eur J Sport Sci. (2021) 1–30. 10.1080/17461391.2021.2001576 34720041

[14] van de Schoor FR Aengevaeren VL Hopman MT Oxborough DL George KP Thompson PD. Myocardial fibrosis in athletes. Mayo Clin Proc. (2016) 91:1617–31. 10.1016/j.mayocp.2016.07.012 27720455

[15] Kellman P Arai AE. Cardiac imaging techniques for physicians: late enhancement. J Magn Reson Imaging. (2012) 36:529–42. 10.1002/jmri.23605 22903654 PMC3428749

[16] Zhang CD Xu SL Wang XY Tao LY Zhao W Gao W. Prevalence of myocardial fibrosis in intensive endurance training athletes: a systematic review and meta-analysis. Front Cardiovasc Med. (2020) 7:585692. 10.3389/fcvm.2020.585692 33102537 PMC7545401

[17] Kent M. The Oxford Dictionary of Sports Science and Medicine. Oxford: Oxford University Press (2006).

[18] Margulis AV Pladevall M Riera-Guardia N Varas-Lorenzo C Hazell L Berkman ND. Quality assessment of observational studies in a drug-safety systematic review, comparison of two tools: the Newcastle-Ottawa Scale and the RTI item bank. Clin Epidemiol. (2014) 6:359–68. 10.2147/CLEP.S66677 25336990 PMC4199858

[19] Abdullah SM Barkley KW Bhella PS Hastings JL Matulevicius S Fujimoto N. Lifelong physical activity regardless of dose is not associated with myocardial fibrosis. Circ Cardiovasc Imaging. (2016) 9:e005511. 10.1161/CIRCIMAGING.116.005511 27903541 PMC5137797

[20] Banks L Altaha MA Yan AT Dorian P Konieczny K Deva DP. Left ventricular fibrosis in middle-age athletes and physically active adults. Med Sci Sports Exerc. (2020) 52:2500–7. 10.1249/MSS.0000000000002411 32472930

[21] Domenech-Ximenos B Sanz-de la Garza M Prat-Gonzalez S Sepulveda-Martinez A Crispi F Duran-Fernandez K. Prevalence and pattern of cardiovascular magnetic resonance late gadolinium enhancement in highly trained endurance athletes. J Cardiovasc Magn Reson. (2020) 22:62. 10.1186/s12968-020-00660-w 32878630 PMC7469354

[22] Merghani A Maestrini V Rosmini S Cox AT Dhutia H Bastiaenan R. Prevalence of subclinical coronary artery disease in masters endurance athletes with a low atherosclerotic risk profile. Circulation. (2017) 136:126–37. 10.1161/CIRCULATIONAHA.116.026964 28465287

[23] Treibel TA Kozor R Menacho K Castelletti S Bulluck H Rosmini S. Left Ventricular hypertrophy revisited: cell and matrix expansion have disease-specific relationships. Circulation. (2017) 136:2519–21. 10.1161/CIRCULATIONAHA.117.029895 29255128

[24] Wilson M O'Hanlon R Prasad S Deighan A Macmillan P Oxborough D. Diverse patterns of myocardial fibrosis in lifelong, veteran endurance athletes. J Appl Physiol (1985). (2011) 110:1622–6. 10.1152/japplphysiol.01280.2010 21330616 PMC3119133

[25] Breuckmann F Mohlenkamp S Nassenstein K Lehmann N Ladd S Schmermund A. Myocardial late gadolinium enhancement: prevalence, pattern, prognostic relevance in marathon runners. Radiology. (2009) 251:50–7. 10.1148/radiol.2511081118 19332846

[26] Malek LA Barczuk-Falecka M Werys K Czajkowska A Mroz A Witek K. Cardiovascular magnetic resonance with parametric mapping in long-term ultra-marathon runners. Eur J Radiol. (2019) 117:89–94. 10.1016/j.ejrad.2019.06.001 31307657

[27] McDiarmid AK Swoboda PP Erhayiem B Lancaster RE Lyall GK Broadbent DA. Athletic cardiac adaptation in males is a consequence of elevated myocyte mass. Circ Cardiovasc Imaging. (2016) 9:e003579. 10.1161/CIRCIMAGING.115.003579 27033835 PMC4841180

[28] Pujadas S Donate M Li CH Merchan S Cabanillas A Alomar X. Myocardial remodelling and tissue characterisation by cardiovascular magnetic resonance (CMR) in endurance athletes. BMJ Open Sport Exerc Med. (2018) 4:e000422. 10.1136/bmjsem-2018-000422 30498573 PMC6241997

[29] Sanchis-Gomar F Lopez-Ramon M Alis R Garatachea N Pareja-Galeano H Santos-Lozano A. No evidence of adverse cardiac remodeling in former elite endurance athletes. Int J Cardiol. (2016) 222:171–7. 10.1016/j.ijcard.2016.07.197 27494731

[30] Swoboda PP McDiarmid AK Erhayiem B Broadbent DA Dobson LE Garg P. Assessing myocardial extracellular volume by T1 mapping to distinguish hypertrophic cardiomyopathy from athlete's heart. J Am Coll Cardiol. (2016) 67:2189–90. 10.1016/j.jacc.2016.02.054 27151352

[31] Tahir E Starekova J Muellerleile K von Stritzky A Munch J Avanesov M. Myocardial fibrosis in competitive triathletes detected by contrast-enhanced CMR correlates with exercise-induced hypertension and competition history. JACC Cardiovasc Imaging. (2018) 11:1260–70. 10.1016/j.jcmg.2017.09.016 29248656

[32] Parry-Williams G Gati S Sharma S. The heart of the ageing endurance athlete: the role of chronic coronary stress. Eur Heart J. (2021) 42:2737–44. 10.1093/eurheartj/ehab095 33748860 PMC8294842

[33] Malek LA Bucciarelli-Ducci C. Myocardial fibrosis in athletes-current perspective. Clin Cardiol. (2020) 43:882–8. 10.1002/clc.23360 32189357 PMC7403702

