# Supplementary material for: The Role of Cardiovascular Magnetic Resonance Imaging in the Assessment of Myocardial Fibrosis in Young and Veteran Athletes: Insights From a Meta-Analysis
Source: Front Cardiovasc Med. 2021 Dec 21;8:784474. doi: 10.3389/fcvm.2021.784474 (PMC8724053; doi:10.3389/fcvm.2021.784474)
Supplement: Supplementary file 1 [file Data_Sheet_1.docx]

Supplementary Material

# Supplementary Figures


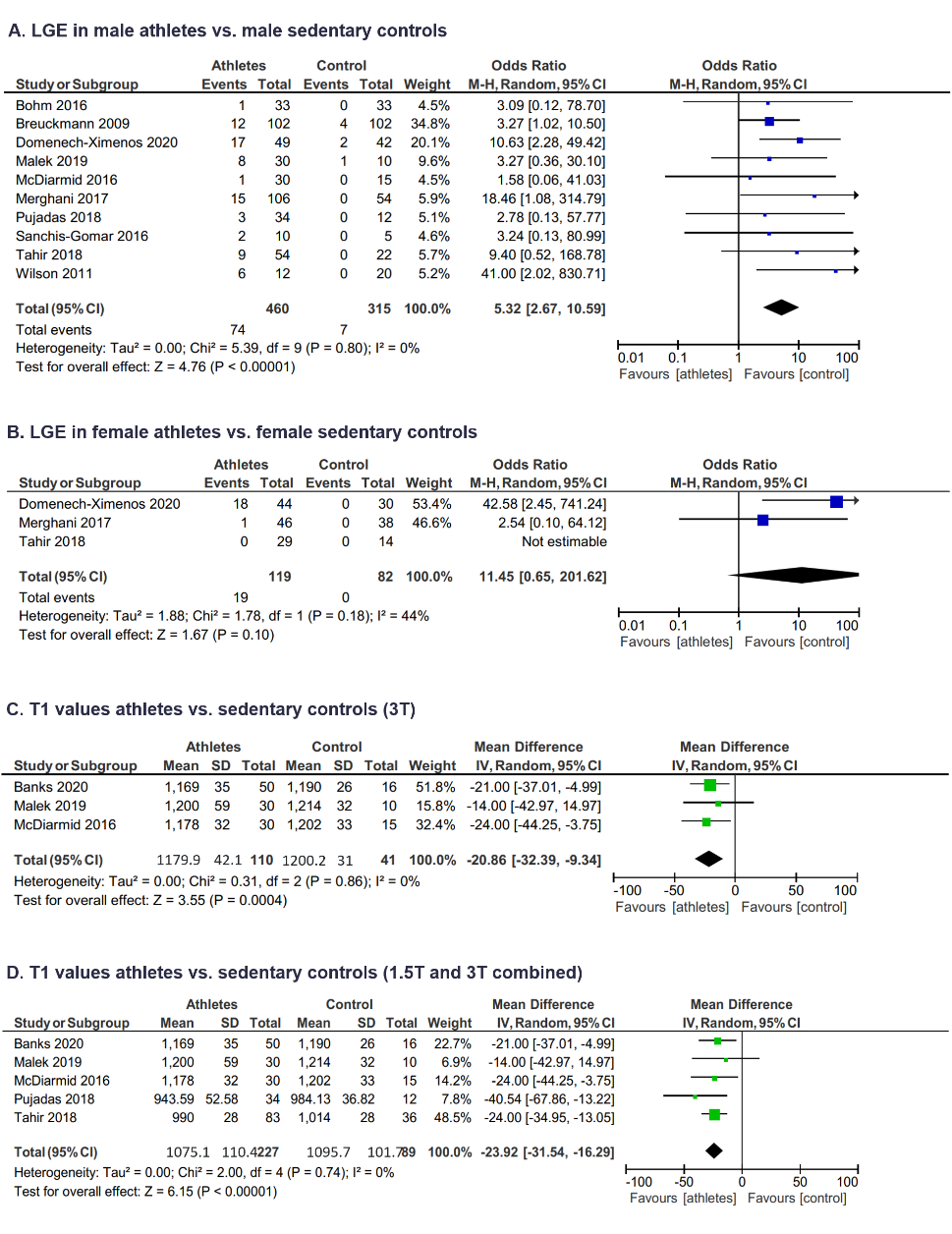


# Supplementary Figure. Forest plots of A) LGE prevalence in male athletes and male sedentary controls, B) LGE prevalence in female athletes and female sedentary controls, C) Native T1 values from 3T CMR scans in athletes and sedentary controls, D) Pooled native T1 values in athletes and sedentary controls

# LGE – late gadolinium enhancement, RV – right ventricle, CMR – cardiac magnetic resonance, ECV – extracellular volume.

# Supplementary Table. Risk of bias assessment via the Newcastle Ottawa Scale for Cohort Studies

|  | **Selection** | | | **Comparability** | **Outcome** | |  |
| --- | --- | --- | --- | --- | --- | --- | --- |
| **Study** | Representativeness of athletes (★) | Selection of controls (★) | Ascertainment of athlete status (★) | (★★) | Assessment of CMR findings (★) | Was duration of intense athleticism enough for outcomes to occur (★) | Total (7★) |
| Abdullah et al. 2016 (13) | ★ | ★ | ★ | - | ★ | ★ | 5 |
| Banks et al. 2020 (14) | ★ | ★ | ★ | - | ★ | ★ | 5 |
| Bohm et al. 2016 (4) | ★ | - | - | ★★ | - | ★ | 4 |
| Breuckman et al. 2009 (19) | ★ | ★ | ★ | ★ | - | ★ | 5 |
| Domenench-Ximenos et al. 2020 (15) | ★ | - | ★ | ★★ | ★ | ★ | 6 |
| Malek et al. 2019 (20) | ★ | - | ★ | ★★ | - | - | 4 |
| Mc Diarmid et al. 2016 (21) | ★ | - | ★ | ★ | ★ | - | 4 |
| Merghani et al. 2017 (16) | ★ | ★ | ★ | - | ★ | ★ | 5 |
| Pujadas et al. 2018 (22) | ★ | - | ★ | ★★ | - | ★ | 5 |
| Sanchis-Gomar et al. 2016 (23) | ★ | - | - | ★★ | ★ | ★ | 5 |
| Swoboda et al. 2016 (24) | ★ | - | ★ | ★★ | - | - | 4 |
| Tahir et al. 2018 (25) | ★ | - | ★ | ★ | ★ | ★ | 5 |
| Treibel et al. 2017 (17) | ★ | - | - | - | - | - | 1 |
| Wilson et al. 2011 (18) | ★ | ★ | ★ | ★ | ★ | ★ | 6 |

# Search strategy

The search process was conducted in accordance with the Preferred Reporting Items for Systematic Reviews and Meta-Analyses (PRISMA) guidelines. A systematic search of relevant studies published up to 01/10/2021 was performed in PubMed, EMBASE and SPORTDiscus. Keywords related to endurance training, late gadolinium enhancement (“LGE”, “late gadolinium enhancement”, “delayed gadolinium enhancement”, “gadolinium”), T1 and extracellular volume (“ECV”) were used as search terms. This study protocol was registered to PROSPERO (ID: CRD42021273996). A.T. and D.M. independently selected which of the search results fulfilled inclusion criteria. In cases of disagreement, it was predetermined that C.B. was going to serve as an arbitrator.

## Inclusion criteria

Publications reporting on outcomes of studies having evaluated one or more of the following parameters in high-performance athletes and sedentary controls:

- The presence of late gadolinium enhancement
- Native T1 values
- Extracellular volume

Only studies recruiting athletes trained in endurance sports were included, using the definition of “a sport that involves continuous high intensity exercise” (Oxford Reference. In The Oxford Dictionary of Sports Science & Medicine (3 ed.) from www.oxfordreference.com/view/10.1093). Only studies reported in English were evaluated for inclusion.

## Exclusion criteria

Studies that didn’t include a control arm were excluded from the systematic review. Studies where either controls or athletes had been included on the basis of having symptoms or signs of cardiac pathology (eg. premature ventricular contractions) were excluded. When multiple included results report on data from the same research group, only one was kept, unless it is explicitly stated that there is no overlap.

## Data extraction

The following data was extracted from each study: Athlete/control age, sex, and characteristics (eg. activity levels, types of sports etc.). Qualitative LGE data were also extracted, with observed prevalence for whole-heart as well as regional-specific LGE data (eg. insertion point only LGE) being recorded for each study. Finally, T1 and ECV values for athletes and controls were also extracted, if available. The synthesis of LGE was performed on the basis of proportions in the athlete and control group, while native T1 (measured in msec) and ECV (reported as a percentage) were synthesized between studies and compared between groups as mean difference. All data were stored in Microsoft Excel datasheets and synthesized with the Review Manager (RevMan) Version 5.3 and SPSS (IBM Corp. Released 2015. IBM SPSS Statistics for Windows, Version 23.0. Armonk, NY: IBM Corp.) software suites.

## PRISMA 2020 flow diagram for updated systematic reviews which included searches of databases, registers and other sources


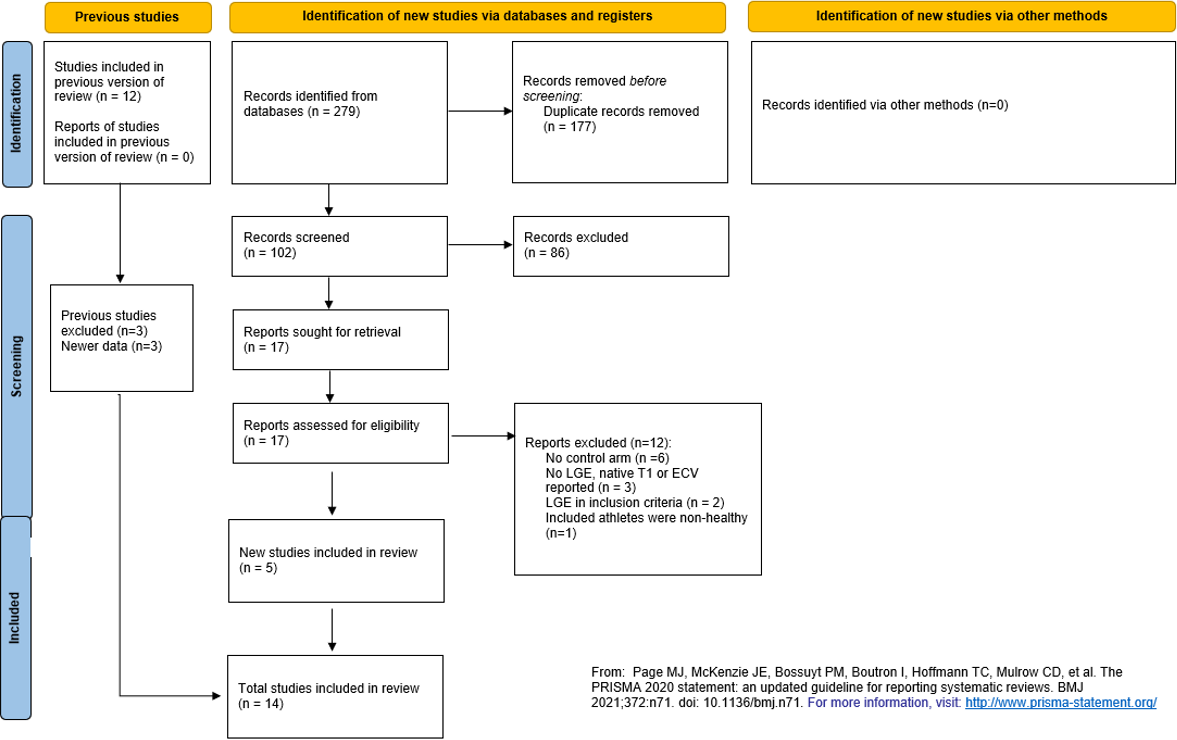


## PRISMA 2020 Checklist


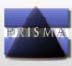
**PRISMA 2020 Checklist**

| **Section and Topic** | **Item #** | **Checklist item** | **Location where item is reported** |
| --- | --- | --- | --- |
| **TITLE** | | |  |
| Title | 1 | Identify the report as a systematic review. | Page 2 |
| **ABSTRACT** | | |  |
| Abstract | 2 | See the PRISMA 2020 for Abstracts checklist. | N/A |
| **INTRODUCTION** | | |  |
| Rationale | 3 | Describe the rationale for the review in the context of existing knowledge. | Page 2 |
| Objectives | 4 | Provide an explicit statement of the objective(s) or question(s) the review addresses. | Page 2 |
| **METHODS** | | |  |
| Eligibility criteria | 5 | Specify the inclusion and exclusion criteria for the review and how studies were grouped for the syntheses. | Page 6, Supplementary Data |
| Information sources | 6 | Specify all databases, registers, websites, organisations, reference lists and other sources searched or consulted to identify studies. Specify the date when each source was last searched or consulted. | Page 2 |
| Search strategy | 7 | Present the full search strategies for all databases, registers and websites, including any filters and limits used. | Page 2, Supplementary Data |
| Selection process | 8 | Specify the methods used to decide whether a study met the inclusion criteria of the review, including how many reviewers screened each record and each report retrieved, whether they worked independently, and if applicable, details of automation tools used in the process. | Page 2, Supplementary Data |
| Data collection process | 9 | Specify the methods used to collect data from reports, including how many reviewers collected data from each report, whether they worked independently, any processes for obtaining or confirming data from study investigators, and if applicable, details of automation tools used in the process. | Supplementary Data |
| Data items | 10a | List and define all outcomes for which data were sought. Specify whether all results that were compatible with each outcome domain in each study were sought (e.g. for all measures, time points, analyses), and if not, the methods used to decide which results to collect. | Supplementary Data |
|  | 10b | List and define all other variables for which data were sought (e.g. participant and intervention characteristics, funding sources). Describe any assumptions made about any missing or unclear information. | Supplementary Data |
| Study risk of bias assessment | 11 | Specify the methods used to assess risk of bias in the included studies, including details of the tool(s) used, how many reviewers assessed each study and whether they worked independently, and if applicable, details of automation tools used in the process. | Page 6 |
| Effect measures | 12 | Specify for each outcome the effect measure(s) (e.g. risk ratio, mean difference) used in the synthesis or presentation of results. | Supplementary Data |
| Synthesis methods | 13a | Describe the processes used to decide which studies were eligible for each synthesis (e.g. tabulating the study intervention characteristics and comparing against the planned groups for each synthesis (item #5)). | Page 6, Supplementary Data |
|  | 13b | Describe any methods required to prepare the data for presentation or synthesis, such as handling of missing summary statistics, or data conversions. | Page 6, Supplementary Data |
|  | 13c | Describe any methods used to tabulate or visually display results of individual studies and syntheses. | Supplementary Data |
|  | 13d | Describe any methods used to synthesize results and provide a rationale for the choice(s). If meta-analysis was performed, describe the model(s), method(s) to identify the presence and extent of statistical heterogeneity, and software package(s) used. | Page 6, Supplementary Data |
|  | 13e | Describe any methods used to explore possible causes of heterogeneity among study results (e.g. subgroup analysis, meta-regression). | Page 6 |
|  | 13f | Describe any sensitivity analyses conducted to assess robustness of the synthesized results. | Page 6 |
| Reporting bias assessment | 14 | Describe any methods used to assess risk of bias due to missing results in a synthesis (arising from reporting biases). | Supplementary Table 2 |
| Certainty assessment | 15 | Describe any methods used to assess certainty (or confidence) in the body of evidence for an outcome. | N/A |
| **RESULTS** | | |  |
| Study selection | 16a | Describe the results of the search and selection process, from the number of records identified in the search to the number of studies included in the review, ideally using a flow diagram. | Page 6, Supplementary Data |
|  | 16b | Cite studies that might appear to meet the inclusion criteria, but which were excluded, and explain why they were excluded. | Supplementary Data |
| Study characteristics | 17 | Cite each included study and present its characteristics. | Page 6, Supplementary Data |
| Risk of bias in studies | 18 | Present assessments of risk of bias for each included study. | Supplementary Table 2 |
| Results of individual studies | 19 | For all outcomes, present, for each study: (a) summary statistics for each group (where appropriate) and (b) an effect estimate and its precision (e.g. confidence/credible interval), ideally using structured tables or plots. | Page 6, Supplementary Table 1 |
| Results of syntheses | 20a | For each synthesis, briefly summarise the characteristics and risk of bias among contributing studies. | Page 6 |
|  | 20b | Present results of all statistical syntheses conducted. If meta-analysis was done, present for each the summary estimate and its precision (e.g. confidence/credible interval) and measures of statistical heterogeneity. If comparing groups, describe the direction of the effect. | Page 4-5 Supplementary Table 1, Figure 1, Supplementary Figure |
|  | 20c | Present results of all investigations of possible causes of heterogeneity among study results. | Figure 1, Supplementary Figure |
|  | 20d | Present results of all sensitivity analyses conducted to assess the robustness of the synthesized results. | Figure 1, Supplementary Figure |
| Reporting biases | 21 | Present assessments of risk of bias due to missing results (arising from reporting biases) for each synthesis assessed. | N/A |
| Certainty of evidence | 22 | Present assessments of certainty (or confidence) in the body of evidence for each outcome assessed. | N/A |
| **DISCUSSION** | | |  |
| Discussion | 23a | Provide a general interpretation of the results in the context of other evidence. | Page 7-8 |
|  | 23b | Discuss any limitations of the evidence included in the review. | Page 7-8 |
|  | 23c | Discuss any limitations of the review processes used. | Page 7-8 |
|  | 23d | Discuss implications of the results for practice, policy, and future research. | Page 7-8 |
| **OTHER INFORMATION** | | |  |
| Registration and protocol | 24a | Provide registration information for the review, including register name and registration number, or state that the review was not registered. | Page 2 |
|  | 24b | Indicate where the review protocol can be accessed, or state that a protocol was not prepared. | Page 2 |
|  | 24c | Describe and explain any amendments to information provided at registration or in the protocol. | Ν/Α |
| Support | 25 | Describe sources of financial or non-financial support for the review, and the role of the funders or sponsors in the review. | Page 8 |
| Competing interests | 26 | Declare any competing interests of review authors. | Page 8 |
| Availability of data, code and other materials | 27 | Report which of the following are publicly available and where they can be found: template data collection forms; data extracted from included studies; data used for all analyses; analytic code; any other materials used in the review. | N/A |

*From:*  Page MJ, McKenzie JE, Bossuyt PM, Boutron I, Hoffmann TC, Mulrow CD, et al. The PRISMA 2020 statement: an updated guideline for reporting systematic reviews. BMJ 2021;372:n71. doi: 10.1136/bmj.n71

For more information, visit: <http://www.prisma-statement.org/>
